# Effect of aquatic physical therapy on chronic low back pain: a systematic review and meta-analysis

**DOI:** 10.1186/s12891-022-05981-8

**Published:** 2022-12-02

**Authors:** Ji Ma, Teng Zhang, Yapeng He, Xin Li, Haoyang Chen, Qian Zhao

**Affiliations:** 1 The Orthopaedic Spinal Ward, Shanxi Provincial People’s Hospital, 29Th Shuangta Temple Street, Taiyuan, Shanxi 030012 People’s Republic of China; 2 School of Nursing, Shanxi Medical University, Yingze District, 56Th Xinjian South Road, Taiyuan, Shanxi 030012 People’s Republic of China; 3 Department of Nursing, Nantong University Affiliated Rehabilitation Hospital, No. 298, Xinhua Road, Nantong, 226000 Jiangsu People’s Republic of China; 4 Department of Nursing, Shanxi Provincial People’s Hospital, 29Th Shuangta Temple Street, Taiyuan, 030012 Shanxi People’s Republic of China

**Keywords:** Chronic low back pain, Aquatic physical therapy, Pain intensity, Quality of life, Disability

## Abstract

**Background:**

Chronic low back pain is a common musculoskeletal disease. With the increasing number of patients, it has become a huge economic and social burden. It is urgent to relieve the burden of patients. There are many common rehabilitation methods, and aquatic physical therapy is one of them. The purpose of this systematic review and meta-analysis is to summarize the existing literature and analyze the impact of aquatic physical therapy on pain intensity, quality of life and disability of patients with chronic low back pain.

**Methods:**

Through 8 databases, we searched randomized controlled trials on the effect of aquatic physical therapy on patients with chronic low back pain. These trials published results on pain intensity, quality of life, and disability. This review is guided by Cochrane Handbook for systematic reviews of interventions version 5.1.0. The level of evidence was assessed through GRADE.

**Results:**

A total of 13 articles involving 597 patients were included. The results showed that compared with the control group, aquatic physical therapy alleviated the pain intensity (Visual Analogue Scale: SMD = -0.68, 95%CI:-0.91 to -0.46, Z = 5.92, *P* < 0.00001) and improved quality of life (physical components of 36-Item Short Form Health Survey or Short-Form 12: SMD = 0.63, 95%CI:0.36 to 0.90, Ζ = 4.57, *P* < 0.00001; mental components of 36-Item Short Form Health Survey or Short-Form 12: SMD = 0.59, 95%CI:0.10 to 1.08, Ζ = 2.35, *P* = 0.02), and reduced disability (Roland Morris Disability Questionnaire: SMD = -0.42, 95%CI:-0.66 to -0.17, Ζ = 3.34, *P* = 0.0008; Oswestry Disability Index or Oswestry Low Back Pain Disability Questionnaire: SMD = -0.54, 95%CI:-1.07 to -0.01, Ζ = 1.99, *P* = 0.05). However, aquatic physical therapy did not improve patients' pain at rest (Visual Analogue Scale at rest: SMD = -0.60, 95%CI:-1.42 to 0.23, Ζ = 1.41, *P* = 0.16). We found very low or low evidence of effects of aquatic physical therapy on pain intensity, quality of life, and disability in patients with chronic low back pain compared with no aquatic physical therapy.

**Conclusions:**

Our systematic review showed that aquatic physical therapy could benefit patients with chronic low back pain. However, because the articles included in this systematic review have high bias risk or are unclear, more high-quality randomized controlled trials are needed to verify.

**Supplementary Information:**

The online version contains supplementary material available at 10.1186/s12891-022-05981-8.

## Background

Chronic low back pain was defined as back pain with or without leg pain for more than 12 weeks between the lower ribs and the folds above the buttocks [1]. Chronic low back pain is a common and increasing skeletal muscle disease [2]. Maher describes back pain syndrome as a major health problem with huge economic and social costs, as more than 80% of health care costs go to patients with the disease [3]. Therefore, it is very important to relieve the pain intensity and disability of patients with chronic low back pain and improve their quality of life.

The treatment of chronic low back pain is still in constant exploration. Scaturro et al. [4] have observed the effect of combination of rehabilitative therapy with ultramized palmitoylethanolamide on patients with chronic low back pain. The results showed that the pain intensity and disability of patients were relieved, and the quality of life was improved. However, Guidelines for the management of patients with chronic low back pain still recommend exercise therapy as a first-line treatment to reduce pain intensity and disability [5]. Among them, aquatic physical therapy is particularly interesting, and one of the methods in rehabilitation treatment recently [6]. Aquatic physical therapy (APT) is defined as exercising in water, or using the characteristics of water to relieve pain intensity, relax muscles and promote better exercise, it includes hydrotherapy and aquatic exercise [7]. Silva et al. previously reported the positive effect of hydrotherapy on the management of patients with knee osteoarthritis [8]. Pérez-de et al. also reported the positive effects of aquatic physical therapy on patients with chronic stroke [9]. Previously, Shi et al. [10] have done a systematic review to analyze the impact of aquatic exercise on patients with chronic low back pain. This article analyzed the impact of aquatic exercise on patients' pain intensity and quality of life. Later, new randomized controlled trials were published, and these articles were not included in the analysis. But in this article, newly published randomized controlled trials was included to analyzed not only the impact of aquatic physical therapy on pain intensity and quality of life of patients with chronic low back pain, but also the impact on disability of patients. Therefore, the purpose of this systematic review is to summarize and analyze the articles about the impact of aquatic physical therapy on patients with chronic low back pain, and to analyze the effectiveness of aquatic physical therapy on pain intensity, quality of life, and disability of patients with chronic low back pain.

## Methods

This systematic review protocol has been registered on PROSPERO as CRD42021265891. The methods was conducted according to the method described in the Cochrane Handbook [11], and the reporting was conducted according to the Preferred Reporting Items for Systematic Review and Meta-Analysis (PRISMA) guidelines [12]. (Appendix 1).

### Search strategy

We searched 8 databases, including: PubMed, Embase, The Cochrane Library, Web of science, China National Knowledge Infrastructure (CNKI), Wanfang data, Chongqing VIP (CQVIP), Chinese Biomedical Literature Database (CBM). There are no restrictions on languages and countries, the search date is from the beginning to July 15, 2022. The following terms were used for retrieval: 'low back pain,' 'aquatic exercise,' 'aquatic therapy,' 'hydrotherapy'.

### Inclusion and exclusion criteria of study

Inclusion and exclusion criteria are based on PICOS standards: see Table 1 for specific inclusion and exclusion criteria.

**Table 1:** Inclusion and exclusion criteria

Category	Inclusion Criteria	Exclusion Criteria
Population	Patients diagnosed with chronic low back pain	Patients with other serious systemic diseases
Interventions	APT or APT + other interventions (APT group)	No APT intervention
Comparisons	Other interventions: such as land based exercise, health education, physical therapy, no exercise, etc. (No APT group)	With APT intervention
Outcomes	Include at least one of the following outcome indicators: pain intensity (VAS, NPRS, NRS etc.); quality of life (SF-36, SF-12 etc.); disability (ODI, ODQ, RMDQ etc.). The analysis results include the short-term (< 12 weeks), medium-term (12–48 weeks) and long-term (> 48 weeks) effects of APT on patients with chronic low back pain	The literature does not contain the outcome indicators of inclusion criteria
Study	Randomized controlled trial; Published in English or Chinese	Non randomized controlled trial

Note: *APT* Aquatic Physical Therapy, *VAS* Visual Analogue Scale, *NPRS* Numeric Pain Rating Scale, *NRS* Numeric Rating Scale, *SF-36* Quality Short-Form 36 Health Survey, *SF-12* Short-Form 12, *ODI* Oswestry Disability Index, *ODQ* Oswestry Low Back Pain Disability Questionnaire, *RMDQ* Roland-Morris Disability Questionnaire

### Study selection

Two researchers independently checked the titles and abstracts of the retrieved studies and downloaded those that might meet the requirements. By reading the full text, the eligible studies were selected according to the inclusion and exclusion criteria. This process was completed by two reviewers, and a third reviewer was sought for discussion to resolve disagreements.

### Data extraction

Two reviewers checked eligible studies and extracted characteristics of included studies, including: the first author, year, country of study, the sample size of the intervention group, the sample size of the control group, the type of exercise in the intervention group, the type of exercise in the control group, intervention time, follow-up time, and outcomes measures.

### Assessment of risk of bias

The quality of the literature was evaluated by two independent people using Cochrane bias risk assessment tool [11] respectively. If there are differences, they should be resolved through discussions with a third reviewer. This tool evaluates the bias of randomized controlled trials in seven aspects: random sequence generation, allocation concealment, blinding of participants and personnel, blinding of outcome assessment, incomplete outcome data, selective reporting and other bias.

### Data synthesis

Meta-analysis was conducted on the same result variable of more than two groups of data. If the mean and standard deviation cannot be directly obtained, the relevant data shall be converted according to the evidence-based medicine conversion formula [13]. The results of data synthesis are presented in the form of forest maps.

### Statistical analysis

We use RevMan (version 5.3) software to analyze the data. Mean ± SD was used as the effect index for continuous variables, 95% confidence intervals (CI) were given for all variables, if the same research tool is used in the included literature, the mean difference (MD) analysis is used. If different research tools are used, the standardized mean difference (SMD) analysis is used. We used Cochran's Q statistic and Ι^2^ statistic to test the heterogeneity of the included articles. If Ι^2^ > 50%, we think there is heterogeneity between articles, and use random effect model for statistical analysis. If Ι^2^ < 50%, the heterogeneity between articles was considered acceptable, and the fixed effect model was used for statistical analysis [14]. Sensitivity analysis and subgroup analysis were used to analyze the source of results heterogeneity. Sensitivity analysis was carried out by eliminating each study one by one. Use Grading of Recommendations Assessment, Development, and Evaluation (GRADE) to rate the quality of evidence as high, moderate, low, or very low. Trials were grouped according to VAS, VAS subscale, SF-36 or SF-12 subscale, RMDQ, ODI, or ODQ.

## Results

### Literature search

**Fig. 1:**
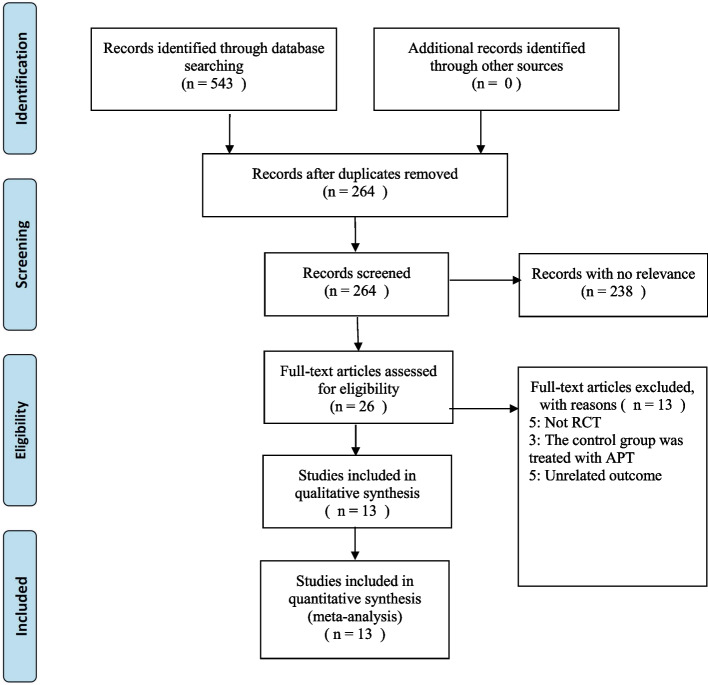
Flow diagram of selection of studies

We used the search strategy to search 543 articles in the database. After excluding the repetitive literatures, we read the titles and abstracts of the remaining literatures, and selected 26 literatures to read the full text. Five non randomized controlled trial was excluded, three in the control group also used aquatic physical therapy, and five articles have unrelated outcome. Finally, 13 articles were confirmed to meet the inclusion criteria. Figure 1 summarizes the literature screening process at the end of the article.

### Study characteristics

This paper includes 13 articles, and the basic characteristics of each article are shown in Table 2.

**Table 2:** Characteristics of included studies

First Author, Year	Country of study	Intervention group(Sample Size)	Control group(Sample Size)	Intervention group (Type of exercise)	Control group (Type of exercise)	Intervention time	Follow up time	Outcomes measures
Abadi et al. 2019 [15]	Sultan	19	20	aquatic exercise	physiotherapy exercise + no exercise	60 min 2 × /wk 12wks	——	modified Oswestry questionnaire
Bello et al. 2010 [16]	Ghana	6	6	water-based exercises	land-based exercises	45-60 min 2 × /wk 6wks	——	Pain: VAS; Trunk flexibility: MSFT,MSET
Costantino et al. 2014 [17]	Italy	27	27	hydrotherapy	physiotherapy treatment	60 min 2 × /wk 12wks	3mons	RMDQ;SF-36
Cuesta-Vargas et al. 2011 [18]	Spain	23	23	MMPTP + DWR	MMPTP	60 min 3 × /wk 15wks	——	pain: VAS; disability: RMDQ; general health:SF-12; physical function: MISL test, dual inclinometer, Biering Sorensen test
Cuesta-Vargas et al. 2012 [19]	Spain	25	24	GP + DWR	GP	30 min 3 × /wk 16wks	48wks	pain: VAS; disability: RMDQ; general health: SF-12
Dundar et al. 2009 [20]	Turkey	32	33	aquatic exercise	land-based exercise	60 min 5 × /wk 4wks	12wks	spinal mobility: modified Schober test; lumbal flexion/extension and rotation: inclinometer, goniometer; pain: VAS; disability: MOLBDQ; quality of life:SF-36
Han et al. 2011 [21]	Korea	9	10	aquatic exercise	no exercise	50 min 5 × /wk 10wks	——	VAS; muscle strength
Mahfouz et al. 2018 [22]	Egypt	20	20	aquatic therapy	conventional physical therapy	60 min 6wks	——	pain: VAS; Disability: ODI; lumbar flexion; Lumbar extension
Nardin et al. 2022 [23]	Brazil	20	20	PBM + DWR	PBM	65 min 2 × /wk 4wks	——	IPAQ-SF; ODI; VAS; 6-min walk test cortisol level; creatine kinase levels
Peng et al. 2022 [24]	China	56	57	therapeutic aquatic exercise	Physical therapy modalities	60 min 2 × /wk 12wks	12mons	RMDQ; NRS; SF-36; SAS; SDS; PSQI; PASS; TSK; FABQ
Sawant et al. 2019 [25]	India	15	15	hydrotherapy	conventional therapy	——	——	VAS; MODI
Yalfani et al. 2020 [26]	Iran	12	12	water pilates	mat pilates	75 min 3 × /wk 8wks	——	pain: VAS; disability: ODQ; balance: BBS
Yücesoy et al. 2021 [27]	Turkey	33	33	balneological + home exercise	home exercise	40 min 5 × /wk 2wks	3mons	pain: VAS, ODI; PGA; DGA; FFD; modified Schober test; SF-36

Note: *VAS* Visual Analogue Scale, *MSFT* Modified Schober Flexion Technique, *MSET* Modified Schober Extension Technique, *RMDQ* Roland-Morris Disability Questionnaire, *SF-36* 36-Item Short Form Health Survey, *MMPTP* Multimodal Physical Therapy Program, *DWR* Deep-Water Running, *SF-12* Short-Form 12, *MISL* Maximum Isometric Strength of Lumbar, *GP* General practice, *MOLBDQ* The modifified Oswestry low back disability questionnaire, *ODI* Oswestry Disability Index, *PBM* Photobiomodulation therapy, *IPAQ-SF* International Physical Activity Questionnaire Short Form, *NRS* Numeric Rating Scale, *SAS* Self-Rating Anxiety Scale, *SDS* Zung Self-rating Depression Scale, *PSQI* Pittsburgh Sleep Quality Index, *PASS* Pain Anxiety Symptoms Scale, *TSK* Tampa Scale for Kinesiophobia, *FABQ* Fear-Avoidance Beliefs Questionnaire, *MODI* Modified Oswestry Disability Index, *ODQ* Oswestry Low Back Pain Disability Questionnaire, *BBS* Biodex Balance System, *PGA* Patient’s global assessment, *DGA* physician’s global assessment, *FFD* finger-to-floor distance

### Risk of bias assessment

**Fig. 2:**
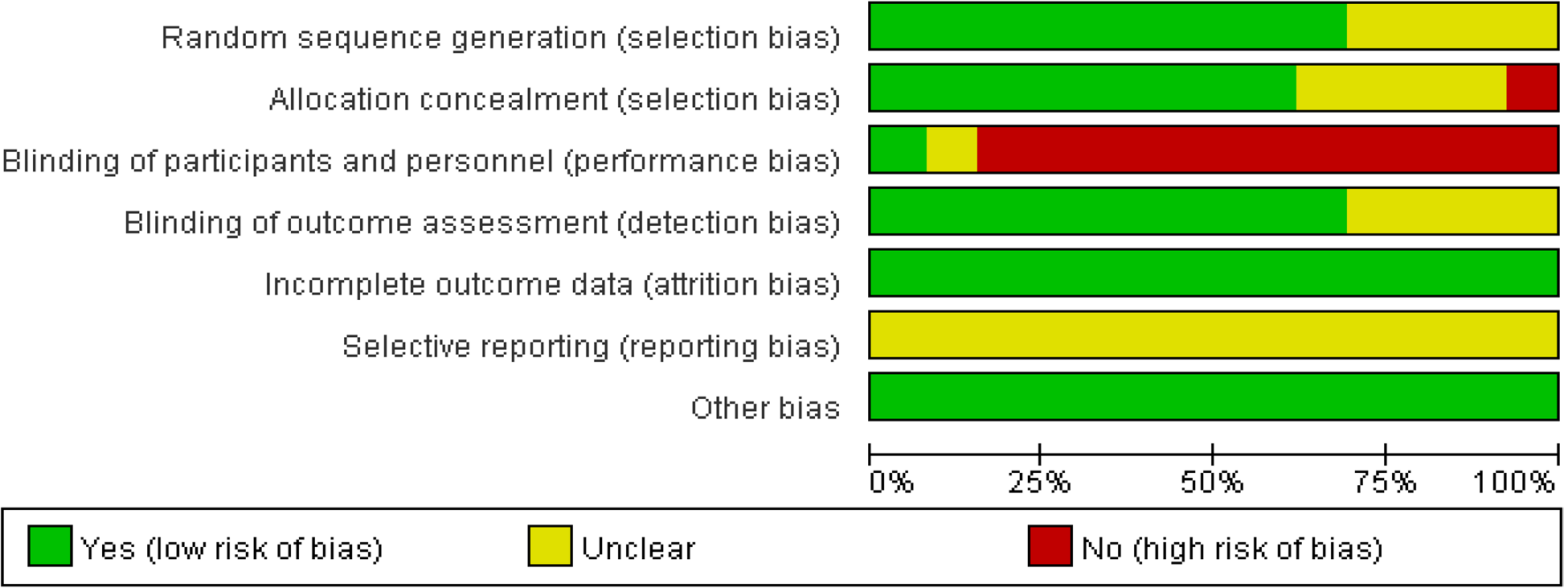
Risk of bias graph

**Fig. 3:**
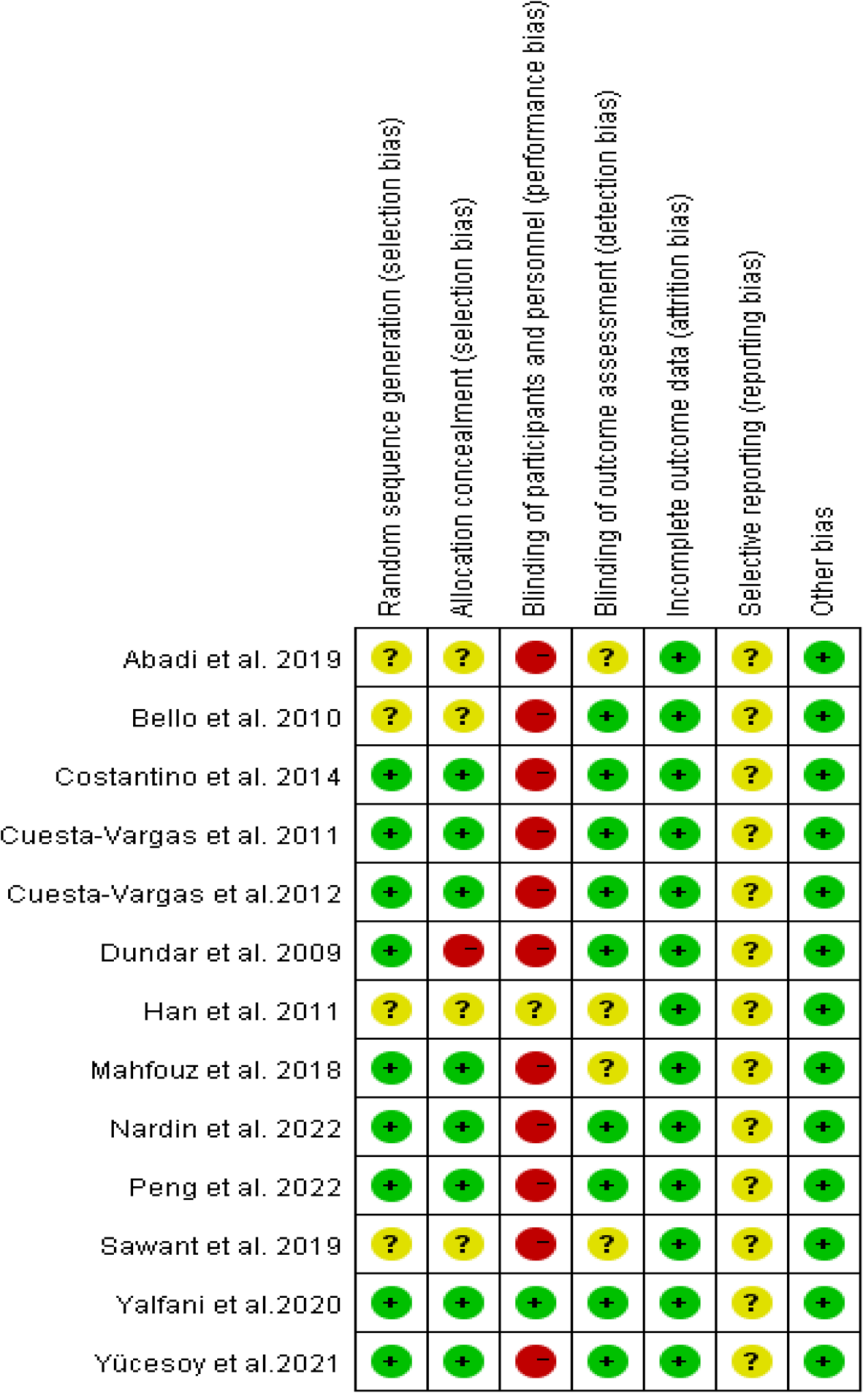
Risk of bias summary

For details of bias risk assessment, see Figs. 2 and 3.

### Effects of interventions

#### APT VS No APT (short-term effects)

**Fig. 4:**
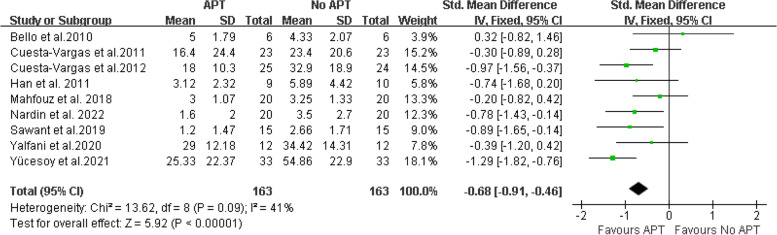
APT VS No APT, VAS

Pain intensity Nine studies [16, 18, 19, 21–23, 25–27] provided data on VAS and were included in the meta-analysis. The final results showed that compared with no aquatic physical therapy, aquatic physical therapy significantly reduced the pain intensity of patients with chronic low back pain (SMD = -0.68, 95%CI:-0.91 to -0.46, Z = 5.92, *P* < 0.00001, Ι^2^ = 41%, Fixed Effect Model). (Fig. 4).

**Fig. 5:**
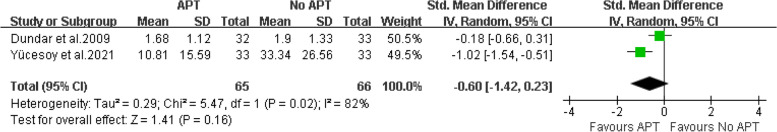
APT VS No APT, VAS at rest

There are two articles [20, 27] that provide data of VAS at rest. The final results showed that compared with no aquatic physical therapy, aquatic physical therapy no significantly reduced the pain intensity of patients with chronic low back pain at rest (SMD = -0.60, 95%CI:-1.42 to 0.23, Ζ = 1.41, *P* = 0.16, Ι^2^ = 82%, Random Effect Model).( Fig. 5).

**Fig. 6:**
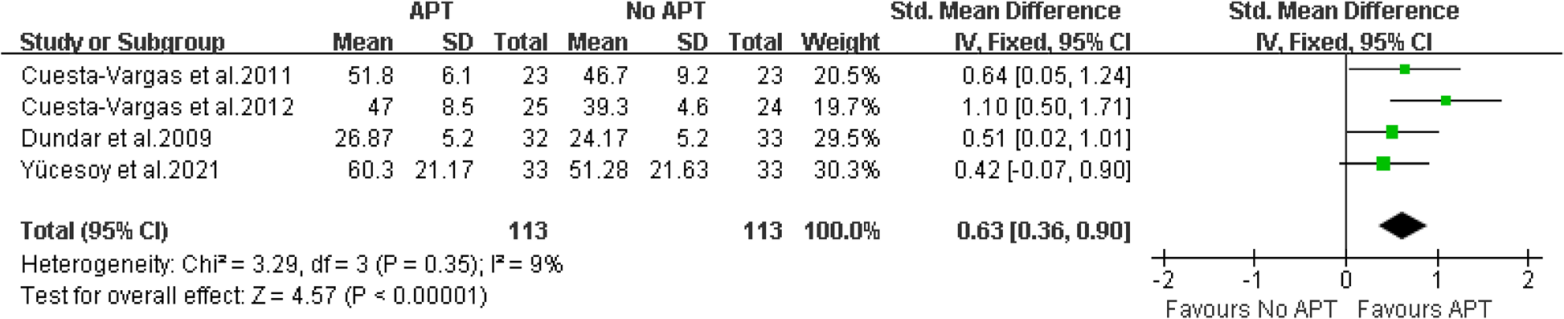
APT VS No APT, physical components of quality of life

Quality of life Four articles [18–20, 27] provide data on the physical components of patients with chronic low back pain treated with aquatic physical therapy. Two articles are provided by SF-36, and two articles are provided by SF-12. Therefore, SMD combined effect quantity is adopted. The final results showed that compared with no aquatic physical therapy, aquatic physical therapy significantly improved the physical condition of patients with chronic low back pain. (SMD = 0.63, 95%CI: 0.36 to 0.90, Ζ = 4.57, *P* < 0.00001, Ι^2^ = 9%, Fixed Effect Model). (Fig. 6).

**Fig. 7:**
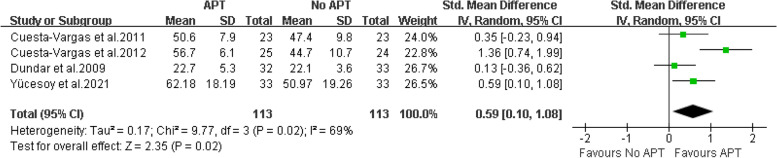
APT VS No APT, mental components of quality of life

Four articles [18–20, 27] provide data on the mental components of patients with chronic low back pain treated with aquatic physical therapy. Two articles are provided by SF-36, and two articles are provided by SF-12. Therefore, SMD combined effect quantity is adopted. Sensitivity analysis found that the heterogeneity decreased from 69% to 0% after deleting one article, this may be due to different assessment tools and different intervention plans [19]. Because the outcome effect was the same, the study by Cuesta-Vargas AI et al. was included in the analysis. The final results showed that compared with no aquatic physical therapy, aquatic physical therapy significantly improved the mental condition of patients with chronic low back pain. (SMD = 0.59, 95%CI: 0.10 to 1.08, Ζ = 2.35, *P* = 0.02, Ι^2^ = 69%, Random Effect Model). (Fig. 7).

**Fig. 8:**
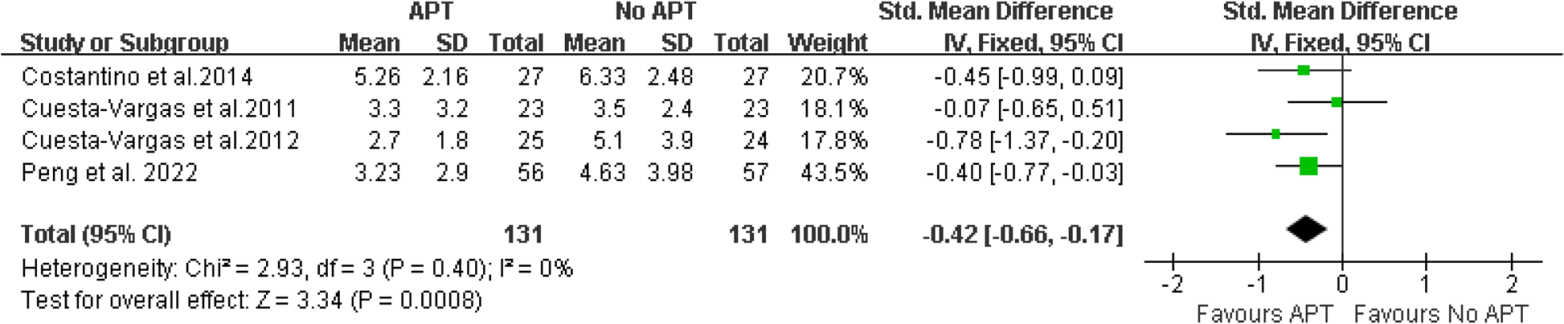
APT VS No APT, RMDQ

Disability Four articles [17–19, 24] provided RMDQ scores. The final results showed that compared with no aquatic physical therapy, aquatic physical therapy significantly improved the disability of patients with chronic low back pain (SMD = -0.42, 95%CI:-0.66 to -0.17, Ζ = 3.34, *P* = 0.0008, Ι^2^ = 0%, Fixed Effect Model). (Fig. 8).

**Fig. 9:**
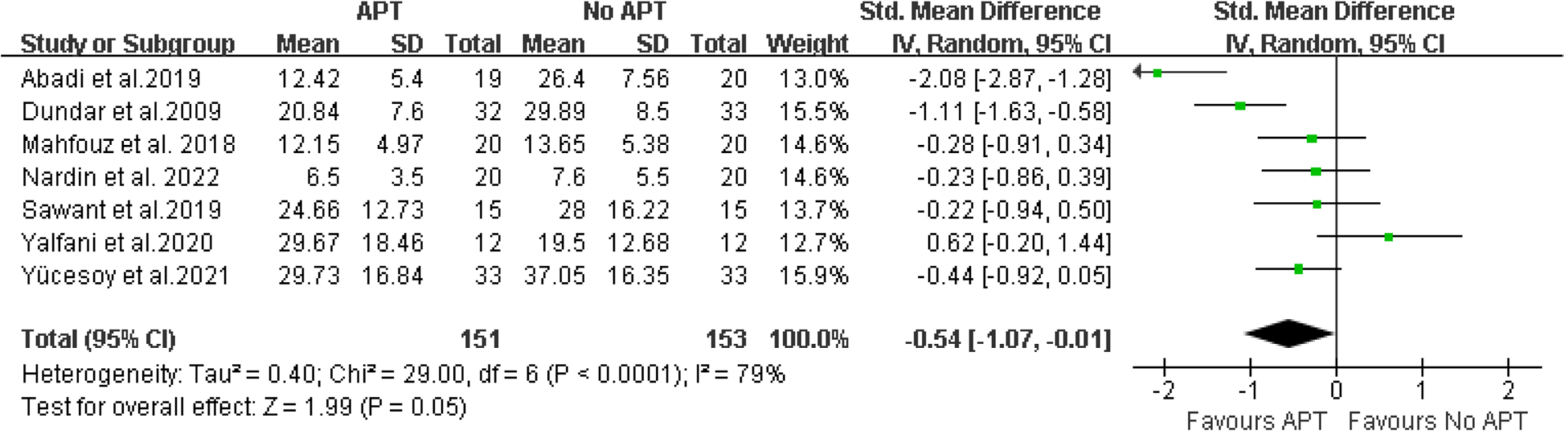
APT VS No APT, ODI or ODQ

Seven articles [15, 20, 22, 23, 25–27] provide ODI or ODQ. The final results showed that compared with no aquatic physical therapy, aquatic physical therapy significantly improved the disability of patients with chronic low back pain. (SMD = -0.54, 95%CI:-1.07 to -0.01, Ζ = 1.99, *P* = 0.05, Ι^2^ = 79%, Random Effect Model) (Fig. 9).

#### Subgroup analysis (APT VS No APT at follow, medium-term effects)

**Fig. 10:**
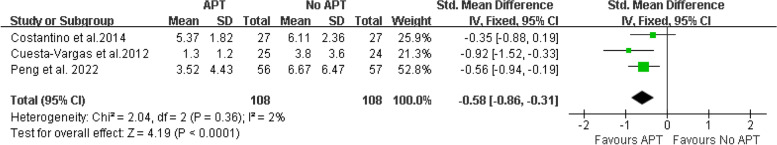
APT VS No APT, RMDQ at follow

Disability Three articles [17, 19, 24] provided RMDQ scores at follow. At follow, compared with no aquatic physical therapy, aquatic physical therapy significantly improved the disability of patients with chronic low back pain (SMD = -0.58, 95%CI:-0.86 to -0.31, Ζ = 4.19, *P* < 0.0001, Ι^2^ = 2%, Fixed Effect Model). (Fig. 10).

**Fig. 11:**
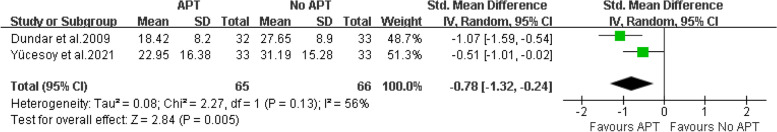
APT VS No APT, ODI or ODQ at follow

Two articles [20, 27] provided ODI or ODQ scores at follow. At follow, compared with no aquatic physical therapy, aquatic physical therapy significantly improved the disability of patients with chronic low back pain (SMD = -0.78, 95%CI:-1.32 to -0.24, Ζ = 2.84, *P* = 0.005, Ι^2^ = 56%, Random Effect Model). (Fig. 11).

#### Subgroup analysis (APT VS land based exercise, short-term effects)

**Fig. 12:**
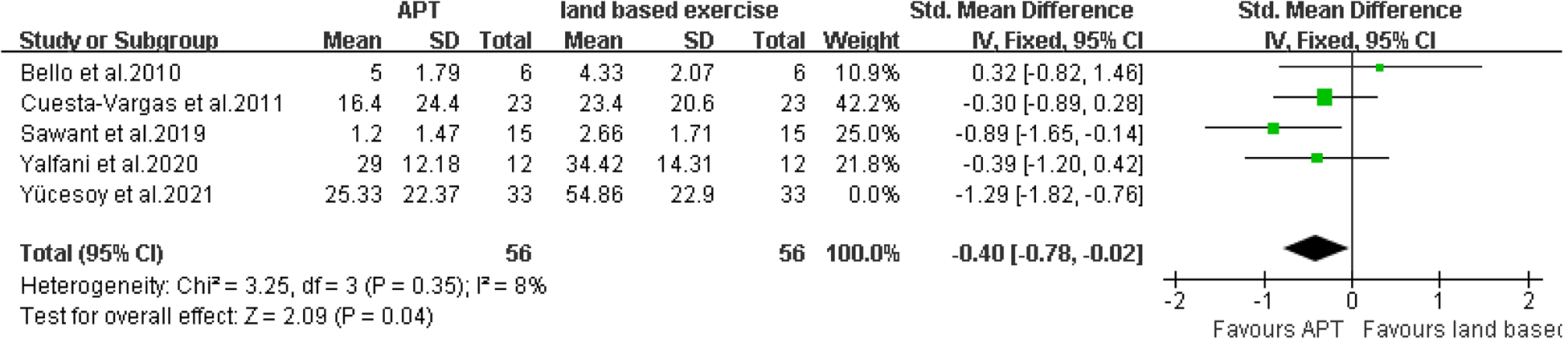
APT VS land based exercise, VAS

Pain intensity There are five articles [16, 18, 25–27] that provided VAS scores. The final results showed that compared with land based exercise, aquatic physical therapy significantly improved the pain intensity of patients with chronic low back pain. (SMD = -0.40, 95%CI:-0.78 to -0.02, Ζ = 2.09, *P* = 0.04, Ι^2^ = 8%, Fixed Effect Model) (Fig. 12).

**Fig. 13:**
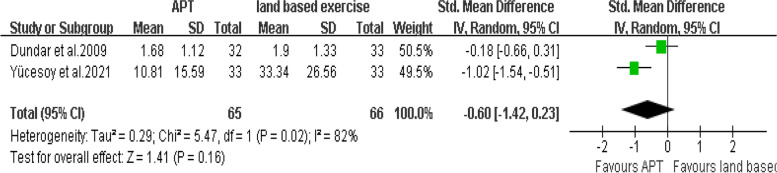
APT VS land based exercise, VAS at rest

There are two articles [20, 27] that provided VAS at rest scores. The final results showed that compared with land based exercise, aquatic physical therapy no significantly improved the pain intensity at rest of patients with chronic low back pain. (SMD = -0.60, 95%CI:-1.42 to 0.23, Ζ = 1.41, *P* = 0.16, Ι^2^ = 82%, Random Effect Model) (Fig. 13).

**Fig. 14:**
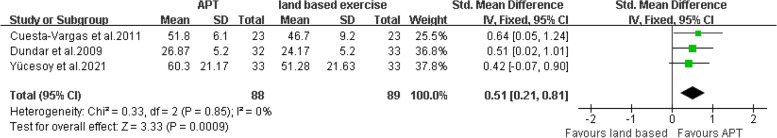
APT VS land based exercise, physical components of quality of life

Quality of life There are three articles [18, 20, 27] that provided physical components scores. The final results showed that compared with land based exercise, aquatic physical therapy significantly improved the physical condition of patients with chronic low back pain. (SMD = 0.51, 95%CI:0. 21 to 0.81, Ζ = 3.33, *P* = 0.0009, Ι^2^ = 0%, Fixed Effect Model) (Fig. 14).

**Fig. 15:**
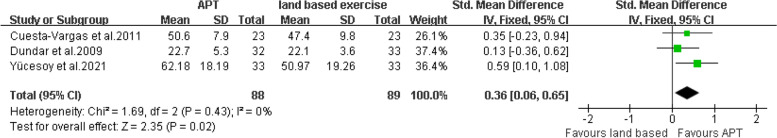
APT VS land based exercise, mental components of quality of life

There are three articles [18, 20, 27] that provided mental components scores. The final results showed that compared with land based exercise, aquatic physical therapy significantly improved the mental condition of patients with chronic low back pain. (SMD = 0.36, 95% CI:0.06 to 0.65, Ζ = 2.35, *P* = 0.02, Ι^2^ = 0%, Fixed Effect Model).( Fig. 15).

**Fig. 16:**
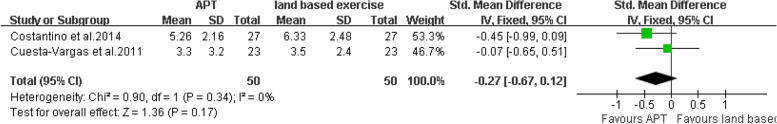
APT VS land based exercise, RMDQ

Disability There are two articles [17, 18] that provided RMDQ scores. The final results showed that compared with land based exercise, aquatic physical therapy no significantly improved the disability of patients with chronic low back pain. (SMD = -0.27, 95%CI:-0.67 to 0.12, Ζ = 1.36, *P* = 0.17, Ι^2^ = 0%, Fixed Effect Model) (Fig. 16).

**Fig. 17:**
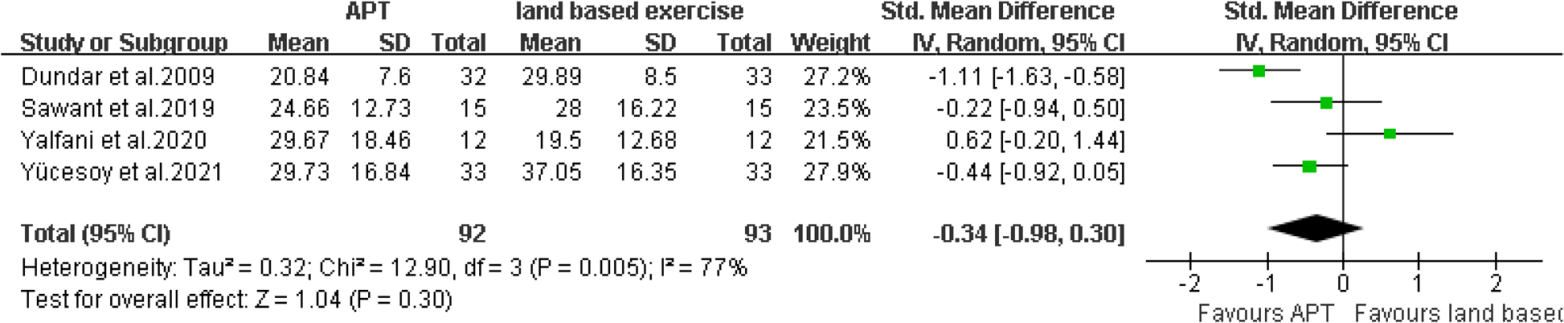
APT VS land based exercise, ODI or ODQ

There are four articles [20, 25–27] that provided ODI or ODQ scores. The final results showed that compared with land based exercise, aquatic physical therapy no significantly improved the disability of patients with chronic low back pain. (SMD = -0.34, 95%CI:-0.98 to 0.30, Ζ = 1.04, *P* = 0.30, Ι^2^ = 77%, Random Effect Model) (Fig. 17).

### Publication bias

As there were no more than 10 included literatures for each outcome, publication bias was not performed.

### Certainty of evidence

For the evidence quality of the measured results, see Table 3 for details.

**Table 3:** GRADE evidence profile of the effect of aquatic physical therapy on chronic low back pain

Certainty assessment							number of patients		Effect	Certainty
Number of studies	Study design	Risk of bias	Inconsistency	Indirectness	Imprecision	Other considerations	APT	No APT	Absolute (95% CI)	
VAS										
9	Randomized trials	serious^1^	not serious^2^	not serious^3^	serious^4^	none^5^	163	163	SMD:-0.68 (-0.91, -0.46)	⊕ ⊕ ◯◯ Low
VAS at rest										
2	Randomized trials	serious^1^	serious^6^	not serious^3^	serious^4^	none^5^	65	66	SMD: -0.60 (-1.42, 0.23)	⊕ ◯◯◯ Very Low
physical components of SF-36 or SF-12										
4	Randomized trials	serious^1^	not serious^7^	not serious^3^	serious^4^	none^5^	113	113	SMD: 0.63 (0.36, 0.90)	⊕ ⊕ ◯◯ Low
mental components of SF-36 or SF-12										
4	Randomized trials	serious^1^	serious^8^	not serious^3^	serious^4^	none^5^	113	113	SMD: 0.59 (0.10, 1.08)	⊕ ◯◯◯ Very Low
RMDQ										
4	Randomized trials	serious^1^	not serious^9^	not serious^3^	serious^4^	none^5^	131	131	SMD: -0.42 (-0.66, -0.17)	⊕ ⊕ ◯◯ Low
ODI or ODQ										
7	Randomized trials	serious^1^	serious^1^^0^	not serious^3^	serious^4^	none^5^	151	153	SMD: -0.54 (-1.07, -0.01)	⊕ ◯◯◯ Very Low

*VAS* Visual Analogue Scale, *SF-36* Quality Short-Form 36 Health Survey, *SF-12* Short-Form 12, *RMDQ* Roland-Morris Disability Questionnaire, *ODI* Oswestry Disability Index, *ODQ* Oswestry Low Back Pain Disability Questionnaire

^1^We downgraded the quality of the evidence for risk of bias by one level. All included studies were at high or unclear risk of bias

^2^We did not downgrade for inconsistency, I^2^ = 41% and χ^2^ = 13.62, *P* = 0.09

^3^Although the studies included different types of interventions, we did not downgrade for indirectness

^4^We downgraded the quality of the evidence by one level because the population size was less than 400 people

^5^We did not downgrade publication bias, although we could not reliably assess this category due to the small number of eligible studies

^6^We did downgrade for inconsistency, I^2^ = 82% and χ^2^ = 5.47, *P* = 0.02

^7^We did not downgrade for inconsistency, I^2^ = 9% and χ^2^ = 3.29, *P* = 0.35

^8^We did downgrade for inconsistency, I^2^ = 69% and χ^2^ = 9.77, *P* = 0.02

^9^We did not downgrade for inconsistency, I^2^ = 0% and χ^2^ = 2.93, *P* = 0.40

^10^We did downgrade for inconsistency, I^2^ = 79% and χ^2^ = 29.00, *P* < 0.0001

## Discussion

This meta-analysis summarizes in detail the effects of aquatic physical therapy on pain intensity, quality of life, and disability in patients with chronic low back pain. In this meta-analysis, we included 13 randomized controlled trials. The results showed that compared with no aquatic physical therapy, aquatic physical therapy can reduce pain intensity, improve quality of life and disability of patients in the short-term.

Pain is the main symptom of patients with chronic low back pain. A VAS was used to assess the patient's pain intensity [28]. It was evaluated as an effective, reliable and responsive technique for pain intensity assessment [16]. In this review, the overall results show that, it can be considered that there is statistical difference, and aquatic physical therapy can relieve the pain intensity of patients with chronic low back pain in the short-term. This result was confirmed in subgroup analysis. In addition, another meta-analysis also showed that there was a positive correlation between aquatic physical therapy and pain intensity relief, aquatic physical therapy can significantly reduce the pain intensity of patients with chronic low back pain [10]. There are two studies [20, 27] giving the VAS score at rest. But it not can be considered that aquatic physical therapy can relieve the pain intensity of patients with chronic low back pain at rest in the short-term. In addition, in subgroup analyses, the VAS at rest scores also was not statistically significant for aquatic physical therapy compared with land based exercise. The reason for this result may be that too few studies were included in the analysis, resulting in bias in the results.

The quality of life was assessed by short form 36 health survey or short form 12 health survey. SF-36 or SF-12 can detect the health changes of the general population. It is a simple and cheap method to measure the health results. It is a continuous ruler to detect the health changes. However, in this review, because other aspects of the data can’t be summarized, we only analyzed the effect on the physical component and mental component. On the physical component and mental component, it can be considered that aquatic physical therapy can improve the physical and mental condition of patients with chronic low back pain in the short-term. This result was confirmed in subgroup analysis. Aquatic physical therapy can help patients relieve their physical and psychological burden.

Low back pain has an impact on a patient's disability because pain limits its activity [29]. Disability was assessed by RMDQ, ODI, ODQ. RMDQ, ODI, ODQ are the three most commonly used scales to assess disability in patients with chronic low back pain [30]. In RMDQ, ODI or ODQ, meta-analysis showed that compared with the control group, the intervention group showed statistically significant, and aquatic physical therapy can improve the disability of patients with chronic low back pain in the short-term. This may be because aquatic physical therapy alleviates the pain intensity of patients with chronic low back pain, thus reducing the impact of pain on the disability of patients, thus improving the disability of patients. However, in the comparison between aquatic physical therapy and land based exercise, the disability of patients was not improved, which may be due to the different intervention protocols included in the studies and the low number of included studies. In addition, subgroup analyses showed that aquatic physical therapy improved disability compared with no aquatic physical therapy in the medium-term.

Aquatic physical therapy is often used as a rehabilitation therapy for patients with musculoskeletal diseases [31]. Moreover, previous studies have demonstrated the safety and effectiveness of aquatic physical therapy [32]. And, the current study included trials related to aquatic physical therapy in patients with chronic low back pain. The results also showed that aquatic physical therapy was effective for patients with chronic low back pain. It is proved that aquatic physical therapy can effectively relieve the pain intensity of patients with chronic low back pain, improve the quality of life and functional ability. It may provide reference for medical staff to make exercise plan for patients with chronic low back pain.

This systematic review included the latest studies for meta-analysis, including 13 randomized controlled trials, while the recently published systematic review only included 8 studies. In addition, this systematic review analyzed the short-term and medium-term effects of aquatic physical therapy on patients with chronic low back pain. However, this review still has some limitations. First, some outcome indicators are measured with different research tools, and there may be measurement bias. Secondly, the modified Jadad tool was planned to be used to evaluate the quality of the included studies at the initial registered protocol. Later, we was considered that the Cochrane bias risk assessment tool was domain-based evaluation. In addition, it requires the evaluation results of each risk bias to have specific judgment reasons and achieve transparency [33].Therefore, we finally used the Cochrane bias risk assessment tool to evaluate the quality of the included studies. Thirdly, researchers should explore the long-term effects of aquatic physical therapy on patients with chronic low back pain. Finally, this review cannot determine the best intervention time and intensity of intervention measures.

## Conclusions

Aquatic physical therapy may be effective and safe in improving pain intensity, quality of life and functional ability in patients with chronic low back pain. Aquatic physical therapy can provide some reference for patients with chronic low back pain when making exercise plans, and encourage patients to carry out aquatic physical therapy. However, more high-quality randomized controlled trials are needed to verify the effectiveness and safety of aquatic physical therapy.

## Supplementary Information

**Additional file 1:** **Appendix 1.** PRISMA check list. **Appendix 2.** Search strategy for Pubmed.

## Data Availability

All data generated or analysed during this study are included in this published article.

## Abbreviations

APT: Aquatic physical therapy
No APT: No aquatic physical therapy
CNKI: China National Knowledge Infrastructure
CQVIP: Chongqing VIP
CBM: Chinese Biomedical Literature Database
CI: Confidence intervals
MD: Mean difference
SMD: Standardized mean difference
